# The fragile healthcare system in Lebanon: sounding the alarm about its possible collapse

**DOI:** 10.1186/s13561-023-00435-w

**Published:** 2023-04-04

**Authors:** Elie Bou Sanayeh, Carolla El Chamieh

**Affiliations:** 1 Department of Internal Medicine, American University of Beirut Medical Center, Bliss Street, Riad El Solh Area, P.O. Box: 11-0236, Beirut, Lebanon; 2 The Holy Spirit University of Kaslik, Jounieh, Lebanon

**Keywords:** Financial Crisis, Healthcare System, Shortage of medications, Staffing shortage

## Abstract

Lebanon is currently facing a complex and multifaceted healthcare crisis. The country has been grappling with a severe financial crisis since 2019, which has been compounded by the social unrest, the devastating Beirut blast in 2020, and the ongoing coronavirus pandemic. Additionally, many hospitals in Lebanon are facing significant difficulties following the devaluation of the Lebanese currency, which has made it difficult for them to purchase necessary medical supplies and equipment. This report aims to examine the difficulties faced by hospitals in Lebanon due to these various factors, and to discuss potential solutions to address the crisis.

## Introduction

Lebanon has always upheld a highly respected healthcare system however, hospitals’ administrators are currently facing several challenges in the wake of the ongoing political, social, and economic crises. Since 2019, the country has been grappling with a severe financial crisis that led to the devaluation of the Lebanese pound and to an enormous fall in the real Gross domestic product per capita [1]. This was further exacerbated by the social unrest, the devastating explosion of Lebanon’s main port in 2020, and the ongoing coronavirus pandemic [1, 2]. These challenges are putting a strain on the fragile Lebanese healthcare system and making it difficult for hospitals to keep up with the increasing demand for healthcare services [3]. Our objective in this report is to shed light on the difficulties faced by hospitals in Lebanon, with a special emphasis on some potential solutions that can be adopted to address the crisis.

## Challenges faced by the hospitals secondary to the financial difficulties

Hospitals in Lebanon are struggling financially due to the difficulties in accessing foreign currency to purchase their needs from abroad, the inability to collect payments from insurance companies, the decrease in government funding, and the maldistribution of available funds [1, 4]. Furthermore, the economic crisis has also affected the patients’ capacity to pay for medical care. Many have lost their jobs or have seen their income reduced, making it difficult for them to afford the cost of medical treatment. This has led to an increase in the number of patients who are unable to pay their medical bills, and as a result, hospitals are struggling to collect payment for the services they provide. This has added to the financial difficulties that hospitals are facing, as they are unable to generate enough revenue to cover their costs. Secondary to this financial instability, the resulting main challenges can be divided into three main categories:

### Shortage of medications and medical equipment

The shortage of medications and medical equipment is a rising concern that peaked during the coronavirus-19 era in the Middle East region [1, 4]. In Lebanon, it became one of the most pressing threats that hospitals are facing. This is partly due to the devaluation of the Lebanese currency, which has made it more expensive for hospitals to import these items. Additionally, there are problems with the distribution and storage of the medications due to lack of electricity and fuel in the country. This has led to a shortage of essential medications, such as chemotherapy and antibiotics [1]. This shortage coupled with the lack of funding for essential services, has led to a decrease in the quality of medical care that hospitals are able to provide. At last, the Coronavirus pandemic has further added to the strain on hospitals in Lebanon, as they are struggling to cope with the overcrowding and the rise in the need for resources [2, 3].

### Staffing shortage

Hospitals are struggling to recruit and retain qualified staff who are choosing to leave the country for better job opportunities elsewhere, as their salaries have taken a pitfall and they felt poorly compensated for their efforts [2, 5]. In fact, with the drop in the local currency’s value over the last three years, many of the specialized physicians fled to secure more stable careers in the United States of America or Europe [5]. As well, the ongoing crisis has taken a toll on Lebanese healthcare workers’ mental well-being [5]. With the significant instability, every facet of their life has shifted causing a great deal of stress, burnout, and anxiety with subsequent plausible negative impact on their overall performance [2, 5].

### Hospitals closures

Hospitals are struggling to pay their bills and meet their financial obligations, such as wages and rent, as they are unable to generate enough revenue to cover their costs. To stay financially afloat, some hospitals were forced to reduce the number of staff and services they offer, while others were forced to shut down. This has left patients without access to essential medical services especially in underprivileged areas, where access to medical care is already limited.

## Impact on health services

For the past decades, Lebanon has attracted international patients as it has some of the most brilliant medical minds. However, the abovementioned factors have led to a decrease in the quality of medical care that hospitals are able to provide. This is particularly concerning for patients with serious and life-threatening conditions that require specialized care [1, 4]. Some patients were turned away, with a subsequent significant loss of revenue for hospitals in Lebanon, as patients are forced to seek medical treatment abroad and specifically in neighboring countries. In addition to the struggle faced to provide adequate specialized care, preventive care such as regular check-ups and screening campaigns has also been affected by the lack of resources and staff.

## The root of the problem

The root of the problem is multifactorial, stemming from a combination of political, economic, and social constraints [3]. For the political aspect, Lebanon has a long history of political instability, and the current climate is marked by divisions and a lack of consensus, which has led to a gridlock in government and a lack of effective decision-making [3]. Further, the government is nationally perceived as corrupt, leading to a lack of trust in its institutions and a lack of confidence in its ability to address the country’s problems. Economically, the country is currently experiencing a financial crisis, marked by high inflation, a devaluation of the Lebanese pound, and high levels of unemployment [1]. This crisis has led to a decrease in both the government funding and the purchasing power of the population. Moreover, Lebanon has been impacted by the Syrian civil war [3], which has led to an influx of refugees, putting further strain on the country’s resources and infrastructure [4]. As for the social aspect, Lebanon has several destressing social issues, such as poverty, inequality, and lack of access to basic services. These problems have been exacerbated by the ongoing crisis and have made it difficult for the population to access healthcare, education, and other service.

## Potential solutions

The situation in Lebanon is dire, and the threats faced by our medical system are only expected to amplify. Thus, both the public and private sectors must work together to implement immediate plans of action that tackle the threats.

On a national level, anti-corruption measures such as regular audits and investigations must be applied to increase transparency and ensure that funds are distributed fairly and efficiently. The Ministry of Public Health must also initiate a long-term healthcare plan to improve the overall system. This plan should focus on investing in infrastructure, increasing funding for public hospitals, and allocating a budget for the importation, transportation, and storage of medical supplies and medications. Additionally, the plan should include measures to address the specific challenges that led to the current situation, prevent similar crises from occurring in the future, and ensure that citizens have equal access to quality healthcare.

On a private level, hospitals’ administrators must face these challenges by imposing cost-cutting measures, such as reducing staff, cutting back on non-essential services, and implementing the use of telemedicine consultations. Additionally, they must investigate alternative funding sources, such as partnerships with international organizations and private donations. Both foreign and local donations are needed initially to boost the collapsed healthcare system, create new job opportunities, and stimulate the economic growth. However, the implementation of monetary and fiscal policies is mandatory to guarantee the financial sustainability and restore investors’ confidence. These steps will support the healthcare system so it can function effectively and efficiently. But the implementation of these solutions will not be easy, and it will require comprehensive, coordinated, and sustained efforts from all stakeholders, including the government, the private sector, and the citizens.

## Conclusion

The healthcare crisis in Lebanon is complex and multifaceted. As a result, hospitals are facing significant difficulties, with some being forced to close due to financial constraints. It is crucial to take comprehensive immediate steps to address these challenges, and to implement long-term plans to improve the overall healthcare system in Lebanon and ensure that the population has access to essential medical services.

## Data Availability

Not applicable.
